# Association of Serum 25-Hydroxyvitamin D Deficiency with Risk of Incidence of Disability in Basic Activities of Daily Living in Adults >50 Years of Age

**DOI:** 10.1093/jn/nxaa258

**Published:** 2020-09-16

**Authors:** Mariane M Luiz, Roberta Máximo, Dayane C Oliveira, Paula C Ramírez, Aline F de Souza, Maicon L B Delinocente, Andrew Steptoe, Cesar de Oliveira, Tiago Alexandre

**Affiliations:** Postgraduate Program in Physical Therapy, Federal University of São Carlos, São Carlos, Brazil; Postgraduate Program in Physical Therapy, Federal University of São Carlos, São Carlos, Brazil; Postgraduate Program in Physical Therapy, Federal University of São Carlos, São Carlos, Brazil; Postgraduate Program in Physical Therapy, Federal University of São Carlos, São Carlos, Brazil; School of Physical Therapy, Santander Industrial University, Bucaramanga, Colombia; Postgraduate Program in Physical Therapy, Federal University of São Carlos, São Carlos, Brazil; Postgraduate Program in Gerontology, Federal University of São Carlos, São Carlos, Brazil; Department of Epidemiology and Public Health, University College London, London, United Kingdom; Department of Epidemiology and Public Health, University College London, London, United Kingdom; Postgraduate Program in Physical Therapy, Federal University of São Carlos, São Carlos, Brazil; Postgraduate Program in Gerontology, Federal University of São Carlos, São Carlos, Brazil; Department of Epidemiology and Public Health, University College London, London, United Kingdom; Gerontology Department, Federal University of São Carlos, São Carlos, Brazil

**Keywords:** 25-hydroxyvitamin D, vitamin D, disability, incidence, aging

## Abstract

**Background:**

Vitamin D deficiency compromises muscle function and is related to the etiology of several clinical conditions that can contribute to the development of disability. However, there are few epidemiological studies investigating the association between vitamin D deficiency and the incidence of disability.

**Objectives:**

We aimed to assess whether vitamin D deficiency is associated with the incidence of disability in basic activities of daily living (BADL) and to verify whether there are sex differences in this association.

**Methods:**

A 4-y follow-up study was conducted involving individuals aged 50 y or older who participated in ELSA (English Longitudinal Study of Ageing). The sample consisted of 4814 participants free of disability at baseline according to the modified Katz Index. Vitamin D was assessed by serum 25-hydroxyvitamin D [25(OH)D] concentrations and the participants were classified as sufficient (>50 nmol/L), insufficient (>30 to ≤50 nmol/L), or deficient (≤30 nmol/L). Sociodemographic, behavioral, and clinical characteristics were also investigated. BADL were re-evaluated after 2 and 4 y of follow-up. The report of any difficulty to perform ≥1 BADL was considered as an incident case of disability. Poisson models stratified by sex and controlled for sociodemographic, behavioral, and clinical characteristics were carried out.

**Results:**

After 4-y follow-up, deficient serum 25(OH)D was a risk factor for the incidence of BADL disability in both women (IRR: 1.53; 95% CI: 1.16, 2.03) and men (IRR: 1.44; 95% CI: 1.02, 2.02). However, insufficient serum 25(OH)D was not a risk factor for the incidence of BADL disability in either men or women.

**Conclusions:**

Independently of sex, deficient serum 25(OH)D concentrations were associated with increased risk of incidence of BADL disability in adults >50 y old and should be an additional target of clinical strategies to prevent disability in these populations.

## Introduction

Vitamin D deficiency, assessed using serum 25-hydroxyvitamin D [25(OH)D] concentrations, is a growing health problem globally owing to its high prevalence (1), with ∼25% of the world's older population presenting with this condition (1, 2).

The role of 25(OH)D in osteomineral metabolism is well known. However, the presence of vitamin D receptors (VDRs) in a range of human tissues enabled researchers to identify a systemic action of vitamin D (3, 4). The discovery of VDRs in myocytes made it possible to identify the role of 25(OH)D in muscle metabolism (5, 6), such as the modulation of calcium influx (Ca^2+^) to muscle cells and in myogenesis (7, 8). Thus, low serum 25(OH)D concentrations may result in less uptake of Ca^2+^ in the muscles, which compromises the quality of muscle contraction and leads to the reduction of muscle mass and strength and muscle atrophy (9, 10). In addition to these biological mechanisms, aging decreases the absorption capacity and cutaneous synthesis of 25(OH)D and promotes a reduction in the number of VDRs in muscle cells (11–13). These compromise the musculoskeletal function and may result in adverse outcomes later in life, such as disability (14, 15).

25(OH)D also participates in the regulation of the metabolism of other important systems that maintain body homeostasis, such as the immune (16) and cardiovascular systems (17). Deficient serum 25(OH)D leads to compromised functioning of these systems and, therefore, can predispose to the development of numerous acute and chronic conditions (18), which impair functional capacity and favor the development of disability (19).

Cross-sectional studies have identified an association between deficient serum 25(OH)D and functional disability to perform basic activities of daily living (BADL) in Japanese and Italian populations (15, 20). However, longitudinal studies have not confirmed these results. Analyzing 665 individuals aged 77 y or older for a 3-y follow-up period, Houston et al. (21) found no association between deficient serum 25(OH)D (<50 nmol/L) and the incidence of disability in BADL. Analyzing 1002 women aged 65 y or older for a 3-y follow-up period, Verreault et al. (22) also found no association between deficient serum 25(OH)D (<25 nmol/L) and the incidence of disability regarding activities that utilize the lower and upper limbs, such as walking a quarter of a mile, walking around the room, climbing 10 steps, sitting down and standing up from a chair, lifting the arms over the head, handling and squeezing objects, and lifting and carrying a 10-pound weight.

Considering the conflicting findings between cross-sectional and longitudinal studies investigating the association between serum 25(OH)D status and functional disability, the aims of the present study were to verify whether deficient serum 25(OH)D is a risk factor for the incidence of BADL disability and whether there are sex differences in this association.

## Methods

### Study population

ELSA (English Longitudinal Study of Ageing) is an ongoing panel study of a representative cohort of men and women living in England aged 50 y or older. It was designed as a sister study to the Health and Retirement Study in the United States and is multidisciplinary in orientation, involving the collection of economic, social, psychological, cognitive, health, biological, and genetic data. The study commenced in 2002, and the sample has been followed up every 2 y through personal interviews and with additional nurse visits for the assessment of biomarkers every 4 y. Ethical approval for all waves of ELSA was granted by the London Multicenter Research and Ethics Committee (MREC 2/1/91) and all participants signed the free and informed consent form. Detailed descriptions of the study design and sampling procedures can be found in a previous publication (23).

**FIGURE 1:**
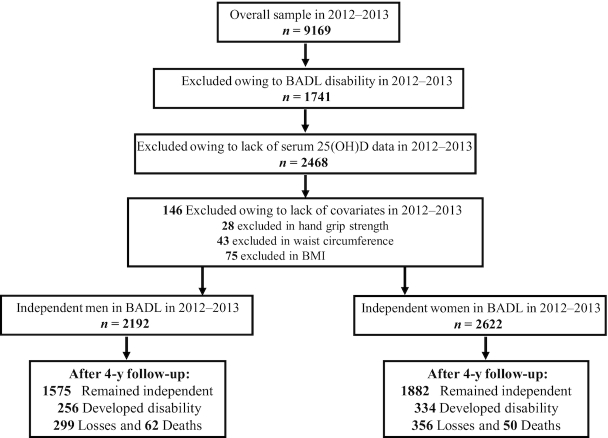
Study design (2012/2013–2016/2017). BADL, basic activities of daily living; 25(OH)D, 25-hydroxyvitamin D.

We analyzed wave 6 (2012–2013) data as our baseline, because this was the first time that serum 25(OH)D concentrations were ascertained in ELSA (24). Out of 9169 participants at baseline, 1741 were excluded owing to having a BADL disability and 2468 owing to missing serum 25(OH)D data. Blood collection was not performed in individuals who had clotting or bleeding disorder, had ever had a seizure, were currently taking anticoagulant drugs, or did not give their consent in writing (24). A further 146 individuals were excluded owing to the lack of information on the covariates. Thus, the final analytical sample at baseline was comprised of 4814 individuals (2192 men and 2622 women) free of BADL disability. The outcome (any incident BADL disability) was assessed at wave 7 (2014–2015) and at wave 8 (2016–2017) (**Figure 1**).

### BADL

BADL were assessed by self-reports of any difficulty in walking, transferring, toileting, bathing, dressing, and eating using the modified Katz Index (25). BADL were assessed at baseline (2012–2013) and reassessed in 2014–2015 and 2016–2017. Only individuals free of any BADL difficulty at baseline were included in the analysis. In 2014–2015 and 2016–2017 all 6 activities were re-evaluated and the BADL outcome was defined: “remained independent for all BADL during the follow-up period” or “developed difficulty to perform one or more BADL during the follow-up period.”

### Serum 25(OH)D

Fasting blood samples were collected for analysis of serum 25(OH)D concentrations. The measurement was performed using chemiluminescent technology (Diasorin Liaison immunoassay) and analyzed at the Royal Victoria Infirmary (Newcastle upon Tyne, United Kingdom). The serum 25(OH)D assay has an analytical sensitivity (lower detection limit) of 7.5 nmol/L. The detection limit represents the lowest measurable analyte concentration that can be distinguished from 0. All assays were performed in duplicate. The CV ranged from 8.7% to 9.4%. The laboratory performing the serum 25(OH)D analyses took part in the Internal and the Vitamin D External Quality Assessment Schemes. Serum 25(OH)D concentrations were used as a continuous variable or categorized as follows: >50 nmol/L = sufficient; >30 and ≤50 nmol/L = insufficient; and ≤30 nmol/L = deficient (26).

### Control variables

The control variables collected at baseline were selected based on previous studies that analyzed factors associated with serum 25(OH)D deficiency (27, 28) and functional BADL disability (29–31).

The sociodemographic variables were age (50–59; 60–69; 70–79; 80–89; 90 y or older), skin color (white; nonwhite), marital status (with or without a conjugal life), schooling (>13 y; 12–13 y; ≤11 y), and wealth (classified in quintiles) (32).

The behavioral characteristics were smoking status (nonsmoker; former smoker; smoker), frequency of alcohol intake (rarely/never; frequently; daily; did not respond), and physical activity (32). Information on physical activity was obtained using 3 questions taken from a validated instrument used in the Health Survey for England on the frequency of physical activities of a mild, moderate, or vigorous intensity; the response options for each category were “more than once a week,” “once a week,” “one to three-time per month,” and “almost never” (33). The individuals were then categorized into 2 groups based on the answers: sedentary (no weekly activity) or active (mild, moderate, or vigorous activity at least once a week) (32).

The clinical conditions were self-reported medical diagnosis of hypertension, diabetes mellitus, cancer, lung disease, heart disease, stroke, osteoporosis, osteoarthritis, dementia, falls in the last year, and hip fractures. The presence of depressive symptoms was recorded when the score on the Center for Epidemiologic Studies—Depression Scale was ≥4 (34).

Waist circumference (WC) was measured with an inextensible metric tape positioned at the midpoint between the last rib and iliac crest with the participant standing, arms alongside the body, trunk bare, and during the expiratory phase. Abdominal obesity was defined as WC >102 cm for men and >88 cm for women (35). The BMI was calculated dividing weight by height squared (kg/m^2^) and classified as follows: <18.5 = underweight; ≥18.5 and <25 = ideal range; ≥25 and <30 = overweight; and ≥30 = obesity (36).

Grip strength was measured using a dynamometer (Smedley's for hand with a scale of 0–100 kg) adjusted to the participant's hand. The maximum strength test was performed 3 times with a 1-min rest period between each trial. Dynapenia was defined as grip strength <26 kg for men and <16 kg for women (37).

The season of the year in which blood was collected for the determination of serum 25(OH)D was recorded and used as a control variable: spring (March–May), summer (June–August), autumn (September–November), and winter (December–February) (38). Vitamin D supplementation and the use of carbamazepine [an anticonvulsant medication with the potential to reduce serum 25(OH)D concentrations] were also used as control variables (39).

### Statistical analysis

The sample characteristics by serum 25(OH)D status at baseline were expressed as means, SDs, and proportions. Differences according to serum 25(OH)D status in individuals free of BADL disability at baseline were analyzed using the chi-square test and ANOVA with Tukey's post hoc test. Differences between included and excluded [owing to the lack of information on serum 25(OH)D and covariables] individuals were analyzed using the chi-square test and Student's *t* test.

Poisson regression models stratified by sex were performed to analyze the association between serum 25(OH)D and the incidence of BADL disability. Firstly, an analysis was performed using serum 25(OH)D as a continuous variable. Subsequently, serum 25(OH)D was used categorically according to the Institute of Medicine cutoff (>50 nmol/L = sufficient; >30 and ≤50 nmol/L = insufficient; and ≤30 nmol/L = deficient) to verify the association of insufficient and deficient serum 25(OH)D with the incidence of BADL disability. The incidence of BADL disability was considered as the development of any difficulty in performing ≥1 BADL during the 4-y follow-up. Control variables with a *P* value ≤ 0.20 in the univariate analyses were incorporated into the multivariate models using the stepwise forward method and those with a *P* value < 0.05 in the final model were considered to be significantly associated with the outcome (40).

To calculate the disability incidence density in each BADL according to serum 25(OH)D status in both sexes, the numerator was the number of individuals who developed disability during the analyzed period and the denominator was the sum of the observation period of the population in question. For individuals who died, the follow-up was computed between the date of the interview in 2012 and the date of death. For individuals who did not develop disability, the follow-up period was computed between the dates of the 2012 and 2016 interviews. For individuals who developed disability, the follow-up period was computed as half of the period between the dates of the 2012 and 2016 interviews. All analyses were performed using the Stata 14 statistical program (Stata Corp.).

## Results

Among the 4814 individuals interviewed and evaluated in 2012, the mean age was 66 y and the majority were women (54.5%), had white skin color, were former smokers, consumed alcohol frequently, and had an active lifestyle. The most prevalent clinical conditions were hypertension (35.3%), osteoarthritis (33.6%), and heart disease (14.4%). The prevalence of insufficient and deficient serum 25(OH)D concentrations was 32% and 23.4%, respectively (**Table 1**).

**TABLE 1:** Sociodemographic, behavioral, and clinical characteristics of 4814 individuals free of BADL disability at baseline according to serum 25(OH)D concentration, ELSA (2012)^1^

	Total (*n* = 4814)	Sufficient^2^ (*n* = 2149)	Insufficient^3^ (*n* = 1538)	Deficient^4^ (*n* = 1127)
Age, y	66.1 ± 8.7	66.1 ± 8.2	66.1 ± 8.7	66.1 ± 9.3
Age, %				
50–59	24.7	23.0	24.5	28.4^a^
60–69	42.2	44.2	42.3	38.4^a^
70–79	25.5	26.6	25.7	23.2
80–89	6.9	5.8	6.8	9.0^a^
≥90	0.7	0.4	0.7	1.0
Sex, %				
Women	54.5	53.7	52.6	58.5^b^
Skin color, %				
Nonwhite	2.6	1.1	2.6^a^	5.6^a,b^
Marital status, %				
With conjugal life	68.6	73.5	67.0^a^	61.5^a,b^
Schooling, %				
>13 y	35.0	36.1	36.0	31.8
12–13 y	29.0	29.1	29.6	28.2
≤11 y	36.0	34.8	34.4	40.0^a,b^
Wealth, %				
Upper quintile	24.7	28.8	24.1^a^	17.7^a,b^
Fourth quintile	23.0	24.4	24.1	18.6^a,b^
Third quintile	21.0	22.1	20.0^a^	20.6^a^
Second quintile	17.6	14.5	18.2^a^	22.5^a^
Lower quintile	11.7	8.5	11.1	18.7^a,b^
Not applicable	2.0	1.7	2.5	1.9
Smoking, %				
Nonsmoker	39.6	40.1	40.6	37.4
Former smoker	49.7	52.3	49.5	44.9^a^
Smoker	10.7	7.6	9.9	17.7
Alcohol intake, %				
Rarely/never	16.8	13.4	16.9^a^	23.1^a,b^
Frequently	40.7	42.2	41.1	37.2^a^
Daily	34.9	39.3	33.7^a^	28.2^a,b^
Not applicable	7.6	5.1	8.3^a^	11.5^a^
Physical activity, %				
Sedentary lifestyle	2.0	1.7	1.8	3.0
Clinical conditions, %				
Hypertension	35.3	33.2	35.6	38.9^a^
Diabetes mellitus	8.4	7.5	8.2	10.3
Cancer	4.9	5.7	4.0	4.6
Heart disease	14.4	14.1	14.4	15.1
Lung disease	12.1	11.5	11.9	13.6
Stroke	2.8	2.4	2.5	3.9
Osteoporosis	6.5	8.6	5.5^a^	3.9^a^
Osteoarthritis	33.6	36.4	32.6	35.6
Dementia	0.4	0.3	0.5	0.7
Falls	18.0	18.9	18.0	16.4
Hip fracture	0.3	0.3	0.3	0.2
Depressive symptoms	8.7	6.8	8.6	12.5^a,b^

1 Continuous variable values are means ± SDs and were compared using ANOVA with Tukey's post hoc test. Categorical variable values are *n* (%) and were compared using the chi-square test. BADL, basic activities of daily living; ELSA, English Longitudinal Study of Ageing; 25(OH)D, 25-hydroxyvitamin D.

2 Serum 25(OH)D concentrations >50 nmol/L.

3 Serum 25(OH)D concentrations >30 to ≤50 nmol/L.

4 Serum 25(OH)D concentrations ≤30 nmol/L.

a Significant difference from sufficient, *P* < 0.05.

b Significant difference from insufficient, *P* < 0.05.

Individuals with deficient serum 25(OH)D had lower levels of schooling and wealth, consumed less alcohol, had a higher prevalence of depressive symptoms, higher waist circumference and BMI, as well as lower grip strength than those with sufficient or insufficient serum 25(OH)D. Moreover, individuals with deficient serum 25(OH)D had a higher prevalence of hypertension and lower prevalence of osteoporosis than those with sufficient serum 25(OH)D (Tables 1, **2**). **Supplemental Tables 1** and **2** show the sample characteristics according to sex.

**TABLE 2:** Anthropometric variables and covariates of 4814 individuals free of BADL disability at baseline according to serum 25(OH)D concentration, ELSA (2012)^1^

	Total (*n* = 4814)	Sufficient^2^ (*n* = 2149)	Insufficient^3^ (*n* = 1538)	Deficient^4^ (*n* = 1127)
Seasonality, %				
Summer	23.2	31.5	20.9^a^	10.1^a,b^
Spring	8.0	5.2	7.8^a^	13.6^a,b^
Autumn	42.4	45.9	44.9	32.6^a,b^
Winter	26.4	17.4	26.4^a^	43.7^a,b^
Vitamin D supplementation, %	4.1	4.2	3.9	4.0
Use of carbamazepine, %	2.0	1.9	2.1	2.0
Waist circumference, cm	94.9 ± 18.5	93.2 ± 23.1	95.7 ± 12.8^a^	97.0 ± 14.4^a,b^
>102 in men; >88 in women, %	47.5	41.0	50.0^a^	56.6^a,b^
BMI, kg/m^2^	27.7 ± 4.8	27.0 ± 4.2	28.0 ± 4.7^a^	28.7 ± 5.5^a,b^
BMI, %				
Ideal range (≥18.5 and <25)	29.0	33.0	26.4^a^	24.9^a^
Underweight (<18.5)	0.8	0.9	0.4	1.2
Overweight (≥25 and <30)	43.5	45.6	44.1	38.7^a,b^
Obese (≥30)	26.7	20.5	29.1^a^	35.2^a,b^
Grip strength, kg	31.6 ± 11.3	32.0 ± 11.4	31.8 ± 11.2	30.7 ± 11.3^a,b^
<26 in men; <16 in women, %	6.7	6.2	6.6	7.8

1 Continuous variables are shown as means ± SDs and were compared using ANOVA with Tukey's post hoc test. Categorical variables are reported as *n* (%) and were compared using the chi-square test. BADL, basic activities of daily living; ELSA, English Longitudinal Study of Ageing; 25(OH)D, 25-hydroxyvitamin D.

2 Serum 25(OH)D concentrations >50 nmol/L.

3 Serum 25(OH)D concentrations >30 to ≤50 nmol/L.

4 Serum 25(OH)D concentrations ≤30 nmol/L.

a Significant difference from sufficient, *P* < 0.05.

b Significant difference from insufficient, *P* < 0.05.

The comparison between individuals included and those excluded owing to missing data in serum 25(OH)D and covariates, but free of disability at baseline, showed that among excluded participants there was a higher proportion of individuals older than 80 y and nonwhite. They also had lower schooling and wealth, smoked more, consumed less alcohol, and were more sedentary. Excluded individuals also had a higher prevalence of hypertension, diabetes mellitus, cancer, heart disease, stroke, and depressive symptoms as well as had a higher waist circumference and lower grip strength than those included (**Supplemental Tables 3, 4**).

In the Poisson models stratified by sex and using serum 25(OH)D concentrations as a continuous variable, we found that the higher the serum 25(OH)D concentration the lower was the risk of disability in women (IRR: 0.99; 95% CI: 0.98, 0.99; *P* = 0.012) but not in men (IRR: 0.99; 95% CI: 0.98, 1.00; *P* = 0.082) (data not shown). In the Poisson models stratified by sex and using serum 25(OH)D divided into 3 different statuses, deficient serum 25(OH)D was independently associated with the incidence of disability in BADL in both sexes. Men and women with deficient serum 25(OH)D had a 44% and 53% higher risk of developing disability in BADL, respectively, than individuals with sufficient serum 25(OH)D (IRR: 1.44; 95% CI: 1.02, 2.02 for men, and IRR: 1.53; 95% CI: 1.16, 2.03 for women). However, insufficient serum 25(OH)D was not a risk factor for the incidence of disability in both sexes (**Table 3**).

**TABLE 3:** Final adjusted Poisson regression models for incidence of disability in ≥1 BADL during a 4-y follow-up in men and women according to serum 25(OH)D concentration, ELSA (2012–2017)^1^

	IRR (95% CI) for incidence of disability in BADL	
Serum 25(OH)D status	Men^2^ (*n* = 1831)	Women^3^ (*n* = 2216)
Sufficient (>50 nmol/L)	1.00	1.00
Insufficient (>30 to ≤50 nmol/L)	1.05 (0.78, 1.41)	1.24 (0.94, 1.62)
Deficient (≤30 nmol/L)	1.44 (1.02, 2.02)	1.53 (1.16, 2.03)

1 Values are IRRs (95% CIs). BADL, basic activities of daily living; ELSA, English Longitudinal Study of Ageing; WC, waist circumference; 25(OH)D, 25-hydroxyvitamin D.

2 Adjusted by age, skin color, schooling, physical activity, smoking, WC, muscle strength, use of carbamazepine, vitamin D supplementation, seasonality, cancer, heart disease, osteoarthritis, falls, hip fracture, and presence of depressive symptoms.

3 Controlled by age, skin color, schooling, smoking, WC, muscle strength, use of carbamazepine, vitamin D supplementation, seasonality, hypertension, lung disease, osteoporosis, osteoarthritis, falls, hip fracture, and presence of depressive symptoms.

**Table 4** shows the disability incidence density in each BADL per 1000 person-years according to the serum 25(OH)D status by sex over the 4-y follow-up. For men who had deficient serum 25(OH)D at baseline, the disability incidence density in bathing, toileting, and walking was statistically higher than for their counterparts with sufficient serum 25(OH)D. For women with deficient serum 25(OH)D at baseline, the disability incidence density in dressing, transferring, bathing, toileting, and walking was statistically higher than for their counterparts with sufficient serum 25(OH)D.

**TABLE 4:** Disability incidence density in each BADL according to serum 25(OH)D concentration and sex, ELSA (2012–2017)^1^

	Disability incidence density per 1000 person-years	
BADL by serum 25(OH)D status	Men	Women
Dressing		
Sufficient^2^	25.8 (21.0, 31.6)	18.8 (15.1, 23.4)
Insufficient^3^	28.2 (22.4, 35.5)	29.5 (23.8, 36.5)^a^
Deficient^4^	34.8 (26.6, 45.5)	39.7 (32.3, 48.8)^a^
Transferring		
Sufficient^2^	22.9 (18.6, 28.2)	24.7 (20.5, 29.8)
Insufficient^3^	24.5 (19.4, 31.1)	34.0 (28.1, 41.1)
Deficient^4^	36.1 (28.1, 46.3)	39.0 (32.0, 47.5)^a^
Bathing		
Sufficient^2^	13.4 (10.2, 17.6)	13.4 (10.4, 17.4)
Insufficient^3^	18.1 (13.8, 23.9)	21.9 (17.2, 27.9)
Deficient^4^	31.1 (23.7, 40.9)^a^	33.0 (26.4, 41.2)^a^
Toileting		
Sufficient^2^	7.1 (4.9, 10.3)	8.6 (6.3, 11.7)
Insufficient^3^	7.2 (4.7, 11.0)	12.8 (9.4, 17.4)
Deficient^4^	17.3 (12.2, 24.6)^a,b^	16.3 (12.1, 22.0)^a^
Walking		
Sufficient^2^	6.5 (4.5, 9.6)	6.7 (4.7, 9.6)
Insufficient^3^	7.9 (5.3, 11.9)	9.5 (6.7, 13.5)
Deficient^4^	14.3 (9.7, 21.0)^a^	15.8 (11.7, 21.4)^a^
Eating		
Sufficient^2^	3.0 (1.7, 5.1)	3.7 (2.3, 5.9)
Insufficient^3^	5.5 (3.3, 8.9)	8.2 (5.6, 11.9)
Deficient^4^	7.0 (4.1, 12.1)	6.3 (3.9, 10.1)

1 Values presented per 1000 person-years (95% CIs). BADL, basic activities of daily living; ELSA, English Longitudinal Study of Ageing; 25(OH)D, 25-hydroxyvitamin D.

2 Serum 25(OH)D concentrations >50 nmol/L.

3 Serum 25(OH)D concentrations >30 to ≤50 nmol/L.

4 Serum 25(OH)D concentrations ≤30 nmol/L.

a Different from sufficient.

b Different from insufficient.

## Discussion

In this large nationally representative sample of older English adults, we found that deficient serum 25(OH)D was a risk factor for the incidence of disability to perform BADL in both sexes, over a 4-y follow-up.

Previous cross-sectional studies have demonstrated an association between deficient serum 25(OH)D and disability (15, 20). However, this association has not been confirmed in longitudinal studies. Houston et al. (21) analyzed 665 individuals and found no association between deficient serum 25(OH)D (<50 nmol/L) and the incidence of disability in BADL over a 3-y follow-up. Verreault et al. (22) analyzed 1002 women during a 3-y follow-up period and also found no association between deficient serum 25(OH)D (<25 nmol/L) and disability.

Some methodological differences between the cited studies and the present investigation may explain the conflicting results. First, we used a cutoff of ≤30 nmol/L to define deficiency, whereas Houston et al. (21) used <50 nmol/L. A cutoff of <50 nmol/L, according to the Institute of Medicine, indicates insufficient serum rather than deficient serum 25(OH)D (26). Thus, higher cutoffs may not be capable of detecting an association with negative outcomes, such as BADL disability. In contrast, Verreault et al. (22), despite using a cutoff of <25 nmol/L, did not find an association with the incidence of disability. This may be explained by the fact that the authors did not exclude individuals with disability at baseline, BADL were not measured using the Katz Index, and the models were not controlled for sociodemographic or behavioral variables.

The role of 25(OH)D in the musculoskeletal system may be one of the main mechanisms by which deficient serum 25(OH)D is a risk factor for the incidence of disability (9, 10). Low serum 25(OH)D concentrations decrease the expression of genes responsible for myogenesis (8, 41) and reduce the synthesis of muscle contractile proteins and the influx of Ca^2+^ into the sarcoplasmic reticulum of muscle cells (9, 10). These biological mechanisms compromise the muscle repair mechanism, alter the kinetics of muscle contraction, and compromise musculoskeletal function. They also lead to decreases in muscle strength and mass and cause atrophy, especially for type II muscle fibers (42–44). Damage to the musculoskeletal system can compromise BADL performance and represent a risk factor for disability.

In addition to the damage to the musculoskeletal system, evidence also shows a relation between serum 25(OH)D concentrations and immunosenescence (45). Deficient serum 25(OH)D is associated with an increased proliferation of inflammatory cytokines by cells of the immune system (46, 47) that leads to low-grade systemic inflammation (44–46). Systemic inflammation plays a crucial role in the etiology of several clinical conditions such as hypertension, diabetes, obesity, and cancer (45) and the presence of multimorbidity can represent an important risk factor for the development of disability (19).

The present study has strengths and limitations that should be considered. A strength is our use of a large representative sample of community-dwelling English individuals aged 50 y or older, which enabled us to perform analyses stratified by sex. Secondly, we included a wide range of socioeconomic, behavioral, and clinical variables to adjust our statistical models. Thirdly, the analysis included data from 3 waves of ELSA, which enabled a reasonably long follow-up time.

Regarding the limitations, disability was evaluated based on self-reports, which could have increased the risk of information bias. However, the Katz Index has international validity and is widely used in studies measuring BADL. ELSA only includes community-dwelling individuals and, therefore, does not allow estimations for institutionalized individuals, who tend to have more BADL disability (30). There was also a dropout rate during follow-up, which, although small, could be a source of bias. However, this occurrence is inevitable in longitudinal studies. Another potential source of bias could be that the majority of individuals we excluded, owing to missing data, were older, were nonwhite, had lower schooling and wealth, smoked more, consumed less alcohol, were more sedentary, had higher prevalence of chronic conditions, as well as had larger WC and lower grip strength than those included. However, it should be pointed out that we found a significant association between deficient serum 25(OH)D and the incidence of disability despite the excluded individuals. Finally, ELSA does not include 2 important control variables that should have been incorporated into the models, namely the parathyroid hormone (PTH) and creatinine concentrations. PTH is high in deficient serum 25(OH)D, characterizing secondary hyperparathyroidism, which is associated with a reduction in strength that could compromise functioning (48, 49). High creatinine concentrations indicate kidney failure, which could interfere with the metabolism of 25(OH)D, contributing to a reduction in its concentrations (50).

In conclusion, deficient serum 25(OH)D was a risk factor for the incidence of BADL disability in both sexes. Therefore, maintaining sufficient concentrations of this vitamin could help prevent the development of disability in individuals aged 50 y and older. Further longitudinal studies are needed to verify deficient serum 25(OH)D as a risk factor for the incidence of disability regarding instrumental activities of daily living.

## Supplementary Material

nxaa258_Supplemental_File

## Notes

Supported by Coordination for the Improvement of Higher Education Personnel – Brazil(CAPES) financing code 001. The English Longitudinal Study of Ageing is financed by Economic and Social Research Council (ESRC)-UK grant 5R01AG017644-16, National Institute on Aging (NIH-USA) grant 5R01AG017644-18, and a consortium of governmental departments of the United Kingdom coordinated by the ESRC. TSA is supported by the Brazilian National Council of Scientific and Technological Development (CNPq) and São Paulo Research Foundation (FAPESP) fund, grants 303981/2017-2 and 2018/13917-3. The funders had no role in the study design, data collection and analysis, or preparation of the manuscript.

Author disclosures: the authors report no conflicts of interest.

Supplemental Tables 1–4 are available from the “Supplementary data” link in the online posting of the article and from the same link in the online table of contents at https://academic.oup.com/jn/.

Abbreviations used: BADL, basic activities of daily living; Ca^2+^, calcium; ELSA, the English Longitudinal Study of Ageing; PTH, parathyroid hormone; VDR, vitamin D receptor; WC, waist circumference; 25(OH)D, 25-hydroxyvitamin D.
