# Supplementary material for: Association of Serum 25-Hydroxyvitamin D Deficiency with Risk of Incidence of Disability in Basic Activities of Daily Living in Adults >50 Years of Age
Source: J Nutr. 2020 Sep 16;150(11):2977–84. doi: 10.1093/jn/nxaa258 (PMC7675030; doi:10.1093/jn/nxaa258)
Supplement: nxaa258_Supplemental_File [file nxaa258_supplemental_file.docx]

**Supplemental Table 1**. Sociodemographic, behavioral, and clinical characteristics of 4,814 individuals free of BADL disability at baseline according to the sex, ELSA Study (2012)^1^

|  | **Total**  **(n = 4,814)** | **Men**  **(n = 2,192)** | **Women**  **(n = 2,622)** |
| --- | --- | --- | --- |
| **Age, years** | 66.1 ± 8.7 | 66.1 ± 8.6 | 66.1 ± 8.7 |
| **Age, %** |  |  |  |
| 50 – 59 years | 24.7 | 24.6 | 24.9 |
| 60 – 69 years | 42.2 | 42.5 | 42.0 |
| 70 – 79 years | 25.5 | 25.4 | 25.7 |
| 80 – 89 years | 6.9 | 6.9 | 6.8 |
| ≥ 90 years | 0.7 | 0.6 | 0.6 |
| **Skin color, %** |  |  |  |
| Non-White | 2.6 | 3.1 | 2.3 |
| **Marital status, %** |  |  |  |
| With conjugal life | 68.6 | 75.9 | 62.6* |
| **Schooling, %** |  |  |  |
| > 13 years | 35.0 | 43.2 | 28.2* |
| 12 to 13 years | 29.0 | 25.7 | 31.8* |
| ≤ 11 years | 36.0 | 31.1 | 40.0* |
| **Wealth, %** |  |  |  |
| Upper quintile | 24.7 | 26.3 | 23.4 |
| 4^th^ quintile | 23.0 | 24.3 | 21.9 |
| 3^rd^ quintile | 21.0 | 21.1 | 21.0 |
| 2^nd^ quintile | 17.6 | 16.0 | 18.9 |
| Lower quintile | 11.7 | 10.4 | 12.8 |
| Not applicable | 2.0 | 1.9 | 2.0 |
| **Smoking, %** |  |  |  |
| Non-smoker | 39.6 | 34.0 | 44.3* |
| Former smoker | 49.7 | 55.6 | 44.7* |
| Smoker | 10.7 | 10.4 | 11.0 |
| **Alcohol intake, %** |  |  |  |
| Rarely/never | 16.8 | 11.2 | 21.5* |
| Frequently | 40.7 | 37.8 | 43.1* |
| Daily | 34.9 | 42.6 | 28.5* |
| Not applicable | 7.6 | 8.4 | 6.9 |
| **Physical activity, %** |  |  |  |
| Sedentary lifestyle | 2.0 | 1.8 | 2.3 |
| **Clinical conditions (yes), %** |  |  |  |
| Hypertension | 35.3 | 37.6 | 33.3* |
| Diabetes mellitus | 8.4 | 10.5 | 6.6* |
| Cancer | 2.6 | 3.1 | 2.3 |
| Heart disease | 14.4 | 16.3 | 12.9* |
| Lung disease | 12.1 | 11.3 | 12.8 |
| Stroke | 2.8 | 3.2 | 2.4 |
| Osteoporosis | 6.5 | 2.0 | 10.3^*^ |
| Osteoarthritis | 33.6 | 26.2 | 39.9^*^ |
| Dementia | 0.4 | 0.7 | 0.2 |
| Falls | 18.0 | 16.0 | 19.7* |
| Hip fractures | 0.3 | 0.3 | 0.2 |
| Depressive symptoms | 8.7 | 6.3 | 10.7* |

^1^ Continuous variables are shown as mean ± SD and compared using the Student t test. Categorical variables are reported as number (%) and compared using the chi-square test.

*Different from men, P < 0.05.

**Supplemental Table 2**. Serum 25(OH)D concentrations, anthropometric variables, and covariates of 4,814 individuals free of BADL disability at baseline according to the sex, ELSA Study (2012)^1^

|  | **Total**  **(n = 4,814)** | **Men**  **(n = 2,192)** | **Women**  **(n = 2,622)** |  |
| --- | --- | --- | --- | --- |
| **Serum 25(OH)D, nmol/L** | 49.6 ± 23.2 | 50.1 ± 23.2 | 49.6 ± 23.2 | |
| **Serum 25(OH)D status, %** |  |  |  | |
| Sufficient (> 50 nmol/L) | 44.6 | 45.4 | 44.1 | |
| Insufficient (> 30 to ≤ 50 nmol/L) | 32.0 | 33.3 | 30.8 | |
| Deficient (≤ 30 nmol/L) | 23.4 | 21.3 | 25.1* | |
| **Seasonality, %** |  |  |  | |
| Spring | 23.2 | 22.5 | 23.6 | |
| Summer | 8.0 | 7.6 | 8.3 | |
| Autumn | 42.4 | 42.7 | 42.3 | |
| Winter | 26.4 | 27.2 | 25.8 | |
| **Vitamin D supplementation (yes), %** | 4.1 | 4.1 | 4.0 | |
| **Use of carbamazepine (yes), %** | 2.0 | 1.9 | 2.1 | |
| **Waist circumference, cm** | 94.9 ± 18.5 | 100.9 ± 22.3 | 90.0 ± 12.5* | |
| >102 men > 88 women, % | 47.5 | 41.0 | 53.0* | |
| **BMI, kg/m^2^** | 27.7 ± 4.8 | 27.8 ± 4.1 | 27.6 ± 5.2 | |
| **BMI, %** |  |  |  | |
| Ideal range (≥ 18.5 and < 25 kg/m^2^) | 29.0 | 24.0 | 33.2* | |
| Underweight (< 18.5 kg/m^2^) | 0.8 | 0.4 | 1.2* | |
| Overweight (≥ 25 and < 30 kg/m) | 43.5 | 50.5 | 37.6* | |
| Obese (≥30 kg/m) | 26.7 | 25.1 | 28.0 | |
| **Grip strength, kg** | 31.6 ± 11.3 | 40.3 ± 9.6 | 24.4 ± 6.5* | |
| <26 men <16 women, % | 6.7 | 5.8 | 7.5 | |

^1^ Continuous variables are shown as mean ± SD and compared using the Student t test. Categorical variables are reported as number (%) and compared using the chi-square test.

*Different from men, P < 0.05.

**Supplemental Table 3**. Sociodemographic, behavioral, and clinical characteristics of individuals included and excluded due to lack on serum 25(OH)D data and covariates but free of BADL disability at baseline, ELSA Study (2012)^1^

|  | **Included**  **(n = 4,814)** | **Excluded**  **(n = 2,614)** |
| --- | --- | --- |
| **Age, years** | 66.1±8.7 | 69.6±10.6 |
| **Age, %** |  |  |
| 50 – 59 years | 24.7 | 23.9 |
| 60 – 69 years | 42.2 | 35.2* |
| 70 – 79 years | 25.5 | 26.4 |
| 80 – 89 years | 6.9 | 12.1* |
| ≥ 90 years | 0.7 | 2.4* |
| **Skin color, %** |  |  |
| Non-white | 2.6 | 5.0* |
| **Marital status, %** |  |  |
| With conjugal life | 68.6 | 64.4* |
| **Schooling, %** |  |  |
| > 13 years | 35.0 | 30.5* |
| 12 to 13 years | 29.0 | 25.1* |
| ≤ 11 years | 36.0 | 44.4* |
| **Wealth, %** |  |  |
| Upper quintile | 24.7 | 21.2* |
| 4^th^ quintile | 23.0 | 20.4 |
| 3^rd^ quintile | 21.0 | 20.5 |
| 2^nd^ quintile | 17.6 | 18.1 |
| Lower quintile | 11.7 | 17.5* |
| Not applicable | 2.0 | 2.3 |
| **Smoking, %** |  |  |
| Non-smoker | 39.6 | 35.1 |
| Former smoker | 49.7 | 49.9 |
| Smoker | 10.7 | 13.0* |
| **Alcohol intake, %** |  |  |
| Rarely/never | 16.8 | 18.1 |
| Frequently | 40.7 | 31.4* |
| Daily | 34.9 | 27.2* |
| Not applicable | 7.6 | 23.3* |
| **Physical activity, %** |  |  |
| Sedentary lifestyle | 2.0 | 5.9* |
| **Clinical conditions (yes), %** |  |  |
| Hypertension | 35.3 | 41.5* |
| Diabetes mellitus | 8.4 | 12.9* |
| Cancer | 4.9 | 7.1* |
| Heart disease | 14.4 | 21.4* |
| Lung disease | 12.1 | 12.9 |
| Stroke | 2.8 | 4.3* |
| Osteoporosis | 6.5 | 6.6 |
| Osteoarthrosis | 33.6 | 31.2 |
| Falls | 18.0 | 18.3 |
| Hip fractures | 0.3 | 0.4 |
| Depressive symptoms | 8.7 | 10.8* |

^1^ Continuous variables are shown as mean ± SD and compared using the Student t test. Categorical variables are reported as number (%) and compared using the chi-square test.

* Different from included, P<0.05.

**Supplemental Table 4**. Anthropometric variables, and covariates of individuals included and excluded due to lack on serum 25(OH)D data and covariates but free of BADL disability at baseline, ELSA Study (2012)^1^

|  | **Included**  **(n = 4,814)** | **Excluded**  **(n = 2,614)** |
| --- | --- | --- |
| **Vitamin D supplementation (yes), %** | 4.1 | 4.6 |
| **Use of carbamazepine (yes), %** | 2.0 | 1.9 |
| **Waist circumference, cm** | 95.9±18.5 | 98.9±25.2* |
| >102 men > 88 women, % | 47.5 | 56.1* |
| **BMI, kg/m^2^** | 27.7±4.8 | 29.1±5.9* |
| **BMI, %** |  |  |
| Ideal range (≥ 18.5 and < 25 kg/m^2^) | 29.0 | 25.8 |
| Underweight (< 18.5 kg/m^2^) | 0.8 | 1.0 |
| Overweight (≥ 25 and < 30 kg/m) | 43.5 | 39.5* |
| Obese (≥30 kg/m) | 26.7 | 33.7* |
| **Grip strength, kg** | 31.6±11.3 | 27.3±9.3* |
| <26 men <16 women, % | 6.7 | 13.3* |

^1^ Continuous variables are shown as mean ± SD and compared using the Student t test. Categorical variables are reported as number (%) and compared using the chi-square test.

* Different from included, P<0.05
